# Functional Properties of Five *Dictyostelium discoideum* P2X Receptors*

**DOI:** 10.1074/jbc.M112.445346

**Published:** 2013-06-05

**Authors:** Abigail Baines, Katie Parkinson, Joan A. Sim, Laricia Bragg, Christopher R. L. Thompson, R. Alan North

**Affiliations:** From the ‡ Faculty of Life Sciences and; § Faculty of Medical and Human Sciences, University of Manchester, Oxford Road, Manchester M13 9PL, United Kingdom

**Keywords:** ATP, Ion Channels, Purinergic Agonists, Purinergic Receptor, Vacuolar Acidification, *Dictyostelium*, Osmoregulation, P2X Receptor

## Abstract

The *Dictyostelium discoideum* genome encodes five proteins that share weak sequence similarity with vertebrate P2X receptors. Unlike vertebrate P2X receptors, these proteins are not expressed on the surface of cells, but populate the tubules and bladders of the contractile vacuole. In this study, we expressed humanized cDNAs of P2XA, P2XB, P2XC, P2XD, and P2XE in human embryonic kidney cells and altered the ionic and proton environment in an attempt to reflect the situation in amoeba. Recording of whole-cell membrane currents showed that four receptors operated as ATP-gated channels (P2XA, P2XB, P2XD, and P2XE). At P2XA receptors, ATP was the only effective agonist of 17 structurally related putative ligands that were tested. Extracellular sodium, compared with potassium, strongly inhibited ATP responses in P2XB, P2XD, and P2XE receptors. Increasing the proton concentration (pH 6.2) accelerated desensitization at P2XA receptors and decreased currents at P2XD receptors, but increased the currents at P2XB and P2XE receptors. *Dictyostelium* lacking P2XA receptors showed impaired regulatory volume decrease in hypotonic solution. This phenotype was readily rescued by overexpression of P2XA and P2XD receptors, partially rescued by P2XB and P2XE receptors, and not rescued by P2XC receptors. The failure of the nonfunctional receptor P2XC to restore the regulatory volume decrease highlights the importance of ATP activation of P2X receptors for a normal response to hypo-osmotic shock, and the weak rescue by P2XB and P2XE receptors indicates that there is limited functional redundancy among *Dictyostelium* P2X receptors.

## Teaser

**Background:** *Dictyostelium discoideum* P2X receptors are found on the contractile vacuole.

**Results:** Four of the five receptors (P2XA, P2XB, P2XD, and P2XE) form ATP-activated channels but differ in their optimal ionic conditions.

**Conclusion:** Properties of five P2X receptors correlate with their rescue of an osmoregulatory phenotype in P2XA-deficient *Dictyostelium* cells.

**Significance:** A P2X receptor is required for normal contractile vacuole operation.

## Introduction

The contractile vacuole system of the social amoeba *Dictyostelium discoideum* is an intracellular organelle comprising interconnected ducts and small cisternae that periodically coalesce into larger intracellular bladders (1). These bladders reach a diameter of 2–4 μm before fusing with the plasma membrane so as to form a pore leading to the extracellular solution (1–3). The contents of the bladder are discharged through this pore, but little or no membrane is exchanged, akin to “kiss and run” exocytosis observed in vertebrate cells. The expulsion of water by this mechanism is considered to be a key component of cell volume regulation when *Dictyostelium* is in a hypo-osmotic solution. The membrane of the empty bladder then fragments and reincorporates into the ducts and cisternae of the contractile vacuole, leading to reformation of the bladder as the cycle repeats. Several of the proteins critical for bladder fusion and emptying have been identified (4–7).

The *D. discoideum* genome encodes five proteins with weak sequence similarity to vertebrate P2X receptors (P2XA, P2XB, P2XC, P2XD, and P2XE) (8). P2X receptors are membrane proteins with an integral ion channel pore that opens as a result of ATP binding. There are seven P2X proteins in most vertebrate species (P2X1–P2X7), and they form functional channels as homo- or heterotrimers. The open channel allows the permeation of small ions, preferably sodium, potassium, and calcium, although some P2X receptors are also permeable to chloride ions and some allow permeation of larger cations such as *N*-methyl-d-glucamine (9, 10). In vertebrate cells, they are localized predominantly in the plasma membrane, and they are activated when ATP binds from the extracellular aspect (9, 10). In contrast, *Dictyostelium* P2X receptors are not found on the plasma membrane, but instead are found inside the cell. Exogenously expressed *Dictyostelium* receptors tagged with green fluorescent protein (GFP) or red fluorescent protein (RFP)^3^ are localized to the contractile vacuole (11, 12). This poses several questions with respect to their functional roles. What is their orientation in the membrane of the contractile vacuole? What is the intracellular source of the ATP? What are the phenotypic consequences of disrupting the expression of one or other P2X receptor? Recent work has begun to address these questions, and significant progress has been made. Sivaramakrishnan and Fountain (13) and Ludlow *et al.* (12) showed that the large cysteine-rich loop that separates the two transmembrane domains and contains the ATP-binding pockets (the “ectodomain” when in a vertebrate cell membrane) is oriented toward the inside of the contractile vacuole. Sivaramakrishnan and Fountain (13) also presented evidence that ATP is translocated from the cytoplasm into the vacuole, and they proposed that this serves to activate the P2X receptor and thus release calcium from the vacuole into the cytoplasm. The phenotypic consequences of disrupting P2X receptor activity on contractile vacuole function have also been investigated. Fountain *et al.* (11) showed that *Dictyostelium* cells lacking a functional P2XA receptor in the AX4 parental strain were impaired in their ability to discharge the contractile vacuole and regulate cell volume when the cells were placed in hypo-osmotic solution, a finding that we have confirmed repeatedly.^4^ Using the AX2 strain, Ludlow *et al.* (12) showed that *Dictyostelium* cells lacking all five receptors could still regulate their cell volume in response to hypo-osmotic stress, although the speed with which the onset of regulatory volume decreases took place was reduced. The difference in severity of the phenotype between the two strains might be partly explained by differences in the magnitude of ATP-evoked calcium release from AX2 and AX4 contractile vacuoles (14). P2X-dependent calcium release appears to be important for osmoregulation in AX4 cells, but is a redundant mechanism in AX2 cells (13, 14).

Despite these advances, several key questions arise from these findings, which are addressed in this study. First, given the weak sequence similarity between P2X receptors in higher organisms and those in *Dictyostelium*, is ATP the preferred or physiological ligand for *Dictyostelium* receptors? This question has recently reasserted itself as it has become clear that some amino acid residues now known to be critically involved in binding the γ-phosphate of ATP (15) are actually not present in most *Dictyostelium* sequences. We approached this by examining the actions of several related nucleotides with known intracellular signaling roles.

Second, are the actions of ATP different in the ionic environment of the contractile vacuole compared with the extracellular solution of vertebrate organisms? Although limited information exists with regard to the ionic composition of the contractile vacuole, it is generally thought to be an acidocalcisome (16). Moreover, it is likely that as water enters and the bladder dilates, ionic concentrations change, although the nature of these changes is poorly understood. We therefore aimed to study in a systematic manner, using heterologous expression, the properties of *D. discoideum* P2X receptors in concentrations of sodium, potassium, and protons that might reflect the situation in living amoeba.

Finally, is there functional redundancy among the different P2X receptors? To address this we have examined the capability of each of the four other receptors to rescue the osmoregulatory defect exhibited by our P2XA deletion strain. Most importantly, we were able to use this system to address whether any such differences in functional response correlated with the properties of the P2X receptor when expressed in a heterologous system.

## EXPERIMENTAL PROCEDURES

### 

#### 

##### Generation and Expression of P2X Constructs

Humanized versions of the P2XB, P2XC, P2XD, and P2XE receptor cDNAs were synthesized by Genscript, as previously described for P2XA receptors (11). These were tagged at the C terminus to express the sequence EYMPME (EE tag) and subcloned into pcDNA3.1 at 5′-HindIII and 3′-XbaI restriction sites. Total protein and membrane expression was measured by Western blotting and biotinylation as described (18). For electrophysiology, human embryonic kidney (HEK) cells were transiently transfected with 1 μg of P2X plasmid and 0.1 μg of enhanced GFP plasmid, using Lipofectamine 2000 (Invitrogen) in accordance with the manufacturer's instructions. For the generation of P2XA-E RFP-tagged overexpression constructs in *Dictyostelium* the coding sequences were amplified by PCR and cloned as translational fusions into the pdm324 plasmid (C-terminal tag of RFP) at BglII and SpeI sites. *Dictyostelium* strains were grown and maintained in association with *Klebsiella aerogenes* or in HL5 axenic medium (17). The RFP-tagged P2X receptors were transformed into the P2XA knock-out background by electroporation and subjected to G418 (20 μg/ml) selection.

##### Electrophysiology

Whole-cell patch recordings were made from HEK293 cells at room temperature (20–23°C), 24–48 h after transfection. Cells were voltage-clamped at −60 mV, and membrane currents were amplified with a HEKA amplifier running Pulse and PULSEFIT software (version 8.54; HEKA). ATP or other potential agonists were applied using an RSC 200 system (Biological Science Instruments) for 2-s duration at 2-min intervals. All data were analyzed using PULSEFIT and Prism (GraphPad) software. Numerical data are presented as mean ± S.E. The significance of differences among groups was assessed by nonparametric analysis of variance and Kruskal-Wallis post hoc tests.

Three intracellular (pipette) solutions were used. NaCl (pH 7.3) contained 145 mm NaCl, 10 mm HEPES, and 10 mm EGTA, and the pH was adjusted to 7.3 with NaOH (final Na^+^ concentration 175 mm). KCl (pH 7.3) contained 145 mm KCl, 10 mm HEPES, and 10 mm EGTA, and the pH was adjusted to 7.3 with KOH (final K^+^ concentration 170 mm). KCl (pH 6.2) contained 145 mm KCl, 10 mm MES, and 10 mm EGTA, and the pH was adjusted to 6.2 with KOH (final K^+^ concentration 165 mm).

Three extracellular solutions were used. NaCl (pH 7.3) contained 147 mm NaCl, 2 mm KCl, 2 mm CaCl_2_, 1 mm MgCl_2_, 13 mm d-glucose, and 10 mm HEPES, and the pH was adjusted to 7.3 with NaOH. KCl (pH 7.3) contained 149 mm KCl, 2 mm CaCl_2_, 1 mm MgCl_2_, 13 mm d-glucose, and 10 mm HEPES, and the pH was adjusted to 7.3 with KOH. KCl (pH 6.2) contained 149 mm KCl, 2 mm CaCl_2_, 1 mm MgCl_2_, 13 mm d-glucose, and 10 mm MES, and the pH was adjusted to 6.2 with KOH. All solutions were maintained at 300–320 mosm/liter. Chemicals were purchased from Sigma.

Concentration-response curves for ATP were generated using either an ascending or a descending order of concentration ([A]). The resulting currents (*I*) were fitted to *I*_max_[A]*^n^*/(EC_50_*^n^* + [A]*^n^*) where *n* is the Hill coefficient. Adenosine and nucleotides were purchased from Sigma.

The effect of copper was assessed for each P2X receptor using the ionic conditions found to give the largest currents in response to ATP. ATP concentrations used were 100 μm (P2XA), 300 μm (P2XE), or 3 mm (P2XB and P2XD) (which correspond to the EC_50_), and percentage inhibition was calculated from currents in the presence and absence of copper (100 nm).

For measurements of calcium permeability it was not possible to use simple bionic solutions because increasing the extracellular calcium concentration strongly inhibited the current evoked by ATP (19). Currents were evoked by ATP (10 μm, rat P2X2; 100 μm, P2XA; 300 μm, P2XB, P2XD, and P2XE) first in 2 mm Ca^2+^ and then in 10 mm Ca^2+^. The internal and external solutions used were those found to provide the maximal ATP currents for the particular receptor (rat P2X2 and P2XA: intracellular NaCl (pH 7.3), extracellular NaCl (pH 7.3); P2XB and P2XE: intracellular KCl (pH 6.2), extracellular KCl (pH 6.2); P2XD: intracellular KCl (pH 6.2), extracellular KCl (pH 7.3)). Reversal potentials were measured from ramp voltage commands (−60 to 60 mV in 500 ms) at peak ATP responses. The reversal potential at 2 mm calcium was arbitrarily set to 0 mV to offset junction potentials, and the change in reversal potential (*E*_r_) observed at 10 mm calcium ([Ca]*_o_*) was then used to compute *P*_Ca_/*P*_Na_ from *P*_Ca_/*P*_Na_ = (1 + exp(*E*_r_*F*/*RT*))·([(γ_K_[K]*_i_* + γ_Na_[Na]*_i_*)(exp(*E*_r_*F*/*RT*)] − [(γ_K_[K]*_i_* + γ_Na_[Na]*_i_*)])/4γ_Ca_[Ca]*_o_* where the activity coefficients γ_Na_, γ_K_, and γ_Ca_ used were 0.75, 0.75, and 0.3, and where *F*, *R*, and *T* have their usual meanings.

##### Immunocytochemistry

48 h after transient transfection, serum was removed from the cells by washing with OptiMEM alone, followed by a 10-min incubation in OptiMEM containing a membrane marker, Alexa Fluor 488 wheat germ agglutinin (5 μg/ml; Invitrogen) in 5% CO_2_/37 °C incubator. Cells were washed once in Hanks' Balanced Salt solution (Invitrogen) and returned to 5% CO_2_/37 °C incubator for a further 20 min. The stained cells were then washed twice in phosphate-buffered saline (PBS), fixed in Zamboni (picric acid and 4%-methanol-free paraformaldehyde) for 10 min at room temperature, washed three times with PBS before permeabilization by incubating in blocking solution (5% goat serum in PBS) containing 0.3% Triton X-100 for 30 min. Cells were then incubated overnight at 4 °C with primary antibody mouse anti-EMPYME (Covance) (1:10,000, in blocking solution). Cells were then washed three times in PBS and incubated for 2 h with Cy3-conjugated goat anti-mouse secondary (1:500) (Jackson Laboratories) at room temperature, washed three times, and mounted using ProLong Gold anti-Fade (Invitrogen). Cells were viewed on an inverted microscope (Nikon Eclipse TE300; Nikon Instruments Europe BV, Amstleven, The Netherlands) equipped with an oil immersion objective (×60, 1.40 N.A.). Image acquisition was controlled with EZ-C1 software, with excitation laser set at either 488 nm (emission of 515 ± 30 nm) for wheat germ agglutinin or 543 nm (emission of 590 ± 50 nm) for P2X receptor staining. Images were processed using Adobe Photoshop and Illustrator SC6 version (Adobe Systems).

##### Rescue of P2XA-Knock-out in Contractile Vacuole Osmoregulation

The P2XA knock-out AX4 strain was available from previous work (11). RFP-tagged P2X receptors were transformed into the P2XA knock-out. After 7 days selection in G418, cells were seeded onto imaging dishes in a solution composed of 16.1 mm KH_2_PO_4_ and 3.7 mm K_2_HPO_4_. Receptors were assayed for their ability to restore the wild type phenotype by placing cells in water for 40 min (this time shows the greatest difference in circularity between wild type knock-out; Ref. 11). At 40 min the circularity of cells was measured from the ratio of two perpendicular axes (circularity index). Rescue is expressed as (1 − circularity index) so that a larger value indicates a more complete rescue. Also at 40 min, the intensity of RFP fluorescence was measured on the same cells. The expression level of RFP differed among cells in the pooled population due to varying copy number, with high copy number cells displaying highest RFP fluorescence. The expression level of RFP was measured in ImageJ (using integrated density measurements) and the value normalized to the cell size (area). Individual cells were the pooled into groups according to expression level (each group contained 4–19 cells). These values were divided by 10,000 to give the arbitrary scale of fluorescence shown on the abscissae in Fig. 6.

The pooled population of transformants was cloned out to give individual clones with either low or high intensity of RFP expression (differing by a factor of 5 in fluorescence). Osmotic shock was performed on these clones by placing them in water for 60 min and measuring circularity at 10-min intervals.

## RESULTS

**FIGURE 1.**
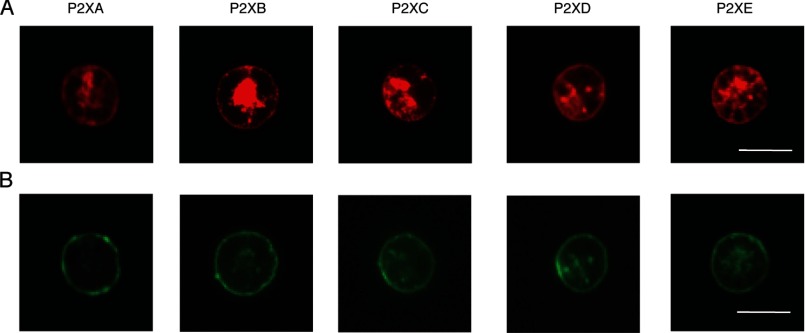
**Expression of P2XA-E receptors in human embryonic kidney cells.** HEK cells were transfected with humanized cDNAs encoding each P2X receptor, with a C terminus EYMPME sequence. An anti-EYMPME antibody was used to detect P2X receptor immunoreactivity (top panels), and Alexa Fluor 488 wheat germ agglutinin to demonstrate membrane labeling (lower panels). All the *Dictyostelium* receptors show expression at plasma membrane by confocal microscopy. Scale bar (10 μm) applies to all panels.

HEK cells showed bright immunofluorescence of the C-terminal EYMPME epitope tag 1–2 days after transfection with humanized cDNAs encoding the each of the five *Dictyostelium* P2X receptors (P2XA–P2XE). In each case, antibody staining was evident at the edge of the cell, corresponding to plasma membrane, as well as in large intracellular pools (Fig. 1). Comparison with the staining of wheat germ agglutinin, used as a cell surface marker, showed membrane labeling of all receptors (Fig. 1). Biotinylation indicated membrane expression of all five *Dictyostelium* constructs. Relative to parallel control studies using rat P2X2 receptors carrying the same epitope, the levels corresponded to 2.5, 2.5, 2.2, 1.2, and 0.7 for P2XA, P2XB, P2XC, P2XD, and P2XE receptors, respectively.

### 

#### 

##### Effect of Extracellular Ions on Responses to ATP

**FIGURE 2.**
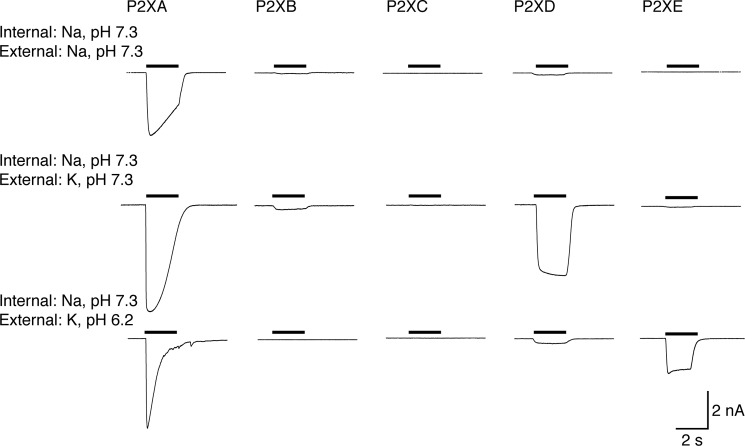
**Changes in extracellular (≈vacuolar) ion concentrations differentially affect P2X receptors.** Each *trace* shows membrane current during application of ATP (3 mm, 2 s: indicated by *bars*). Intracellular (≈cytoplasmic) cation was sodium. P2XA receptors are activated under all conditions. P2XD receptors are activated by ATP when potassium is the external cation, but are inhibited by acidification. P2XE receptors are activated only with external potassium and acidification.

In view of the evidence for the orientation of the P2X receptor in the contractile vacuole (12, 13), the extracellular solution in the electrophysiological experiments would correspond to the intravacuolar solution in amoeba. Under control conditions, with Na^+^ as the predominant extracellular cation, ATP (3 mm) elicited a large inward current at P2XA receptors that partly desensitized during a 2-s application (Fig. 2) (11, 12). P2XB–P2XE receptors did not respond to ATP (3 mm) (Table 1).

**TABLE 1:** **Currents evoked by ATP (3 mm) in HEK cells expressing P2XA–E receptors, under varying internal/external ionic conditions** Currents are in picoamperes/picofarad ± S.E. for the number of cells shown (in parentheses). Asterisks indicate significantly different from Na^+^/Na^+^ condition (*, *p* < 0.05; **, *p* < 0.01; ***, *p* < 0.001).

Internal (pipette) solution	External solution	P2XA	P2XB	P2XC	P2XD	P2XE
NaCl pH 7.3	NaCl pH 7.3	395 ± 39 (19)	<5 (5)	<5 (6)	<5 (5)	<5 (5)
	KCl pH 7.3	544 ± 73 (8)	30 ± 18 (4)	<5 (5)	**203 ± 41 (14)	21 ± 6 (4)
	KCl pH 6.2	494 ± 85 (5)	14 ± 9 (4)	<5 (6)	43 ± 34 (6)	**201 ± 36 (5)
KCl pH 7.3	NaCl pH 7.3	405 ± 107 (5)	21 ± 13 (5)	<5 (4)	20 ± 13 (4)	<5 (5)
	KCl pH 7.3	323 ± 67 (5)	42 ± 18 (3)	<5 (4)	*224 ± 25 (5)	17 ± 9 (5)
	KCl pH 6.2	275 ± 14 (4)	71 ± 32 (3)	0 (3)	184 ± 83 (6)	**178 ± 17 (4)
KCl pH 6.2	NaCl pH 7.3	661 ± 73 (6)	39 ± 24 (3)	<5 (3)	21 ± 9 (4)	<5 (3)
	KCl pH 7.3	394 ± 95 (4)	88 ± 14 (3)	<5 (3)	***312 ± 56 (9)	22 ± 10 (4)
	KCl pH 6.2	268 ± 94 (5)	***148 ± 30 (8)	0 (3)	160 ± 56 (3)	***313 ± 41 (5)

**FIGURE 3.**
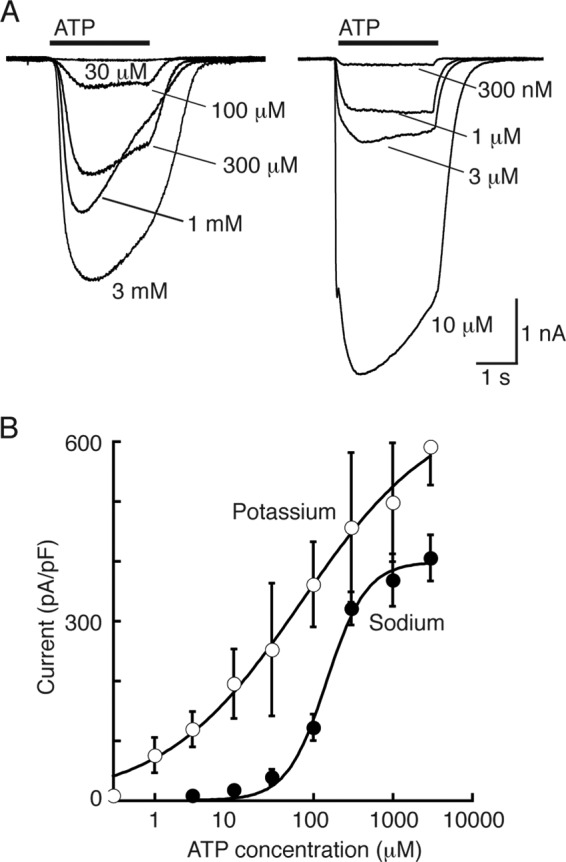
**P2XA receptors are more sensitive to ATP with extracellular (≈vacuolar) potassium ions.** *A*, *left*, currents evoked by ATP (2 s: indicated by *bar*) in external sodium. *A*, *right*, currents evoked by ATP (2 s: indicated by *bar*) in external potassium. Intracellular (≈cytoplasmic) cation was sodium. *B*, concentration-response curves for ATP left-shifted in extracellular potassium.

Substitution of extracellular Na^+^ by K^+^ increased the amplitude of responses to ATP in cells expressing P2XA receptors (Fig. 2). This effect was observed at all ATP concentrations tested and resulted in a shift in the pEC_50_ for ATP from 3.8 ± 0.07 (*n* = 20 or 21) to 4.0 ± 1.2 (*n* = 3–7) (Fig. 3). The effect of K^+^ substitution was particularly marked at low ATP concentrations: 1, 3, and 10 μm ATP were ineffective in extracellular Na^+^, but evoked robust currents in extracellular K^+^ (Fig. 3). The slope of the concentration-response curve was much reduced in K^+^ solutions: *n*_H_ in Na^+^ was 2.0 ± 0.5 (*n* = 20–21), and in K^+^ was 0.35 ± 0.35 (*n* = 3–7). Currents at P2XA receptors desensitized more rapidly in a K^+^-based extracellular solution. The amplitude at the end of a 2-s application as a fraction of peak amplitude was 0.71 ± 0.05 in Na^+^ (*n* = 11) and 0.28 ± 0.10 in K^+^ (*n* = 10). Cells expressing P2XB, P2XC, and P2XE receptors did not respond to ATP with significant currents under these conditions. However, ATP (3 mm) evoked large currents at P2XD receptors (Fig. 2 and Table 1); these currents had a rapid onset and showed minimal desensitization during a 2-s application (amplitude at 2 s was 0.96 ± 0.01 (*n* = 33) of peak amplitude).

Acidification (to pH 6.2) of the K^+^-containing extracellular solution had little effect on the peak currents elicited by ATP in cells expressing P2XA receptors (Fig. 1 and Table 1) other than to further increase their desensitization (fraction of peak current at 2 s was 0.10 ± 0.05 (*n* = 6)). Cells expressing P2XB and P2XC receptors did not respond significantly to ATP. P2XD receptors were strongly inhibited by pH 6.2 (Fig. 2 and Table 1) whereas cells expressing P2XE receptors showed currents in response to ATP that were not observed at pH 7.3 (Fig. 2 and Table 1). These currents showed little desensitization (amplitude at 2 s was 0.91 ± 0.03 (*n* = 24) of peak amplitude).

The relative permeability to calcium ions (*P*_Ca_/*P*_Na_) is ideally measured in bi-ionic conditions (calcium outside, sodium inside). However, increasing the extracellular calcium concentration strongly inhibited ATP-evoked currents at all *Dictyostelium* P2X receptors (19). We therefore estimated *P*_Ca_/*P*_Na_ by measuring the change in reversal potential observed when [Ca]*_o_* was changed from 2 to 10 mm (see “Experimental Procedures”). Under these conditions, P2XB and P2XE receptors showed no detectable calcium permeability. P2XA and P2XD receptors gave an “apparent” *P*_Ca_/*P*_Na_ of 3.2 ± 1.2 (*n* = 13) and 0.74 ± 0.18 (*n* = 14), respectively, whereas the value for rat P2X2 receptors under the same conditions was 15.0 ± 2.0 (*n* = 12). Although these values cannot be compared directly with estimates made under bi-ionic solutions, they indicate that under conditions that provide optimal responses to ATP they are considerably less permeable to calcium than are rat P2X2 receptors.

*Dictyostelium* P2XA receptors are particularly sensitive to block by extracellular copper (11). In the present work we measured the percentage inhibition of the ATP-evoked current by 100 nm copper: these values were: P2XA, 44 ± 5.8 (*n* = 9); P2XB, 84 ± 4.2 (*n* = 15); P2XD, 30 ± 3.3 (*n* = 9); and P2XE, 70 ± 9.0 (*n* = 9).

##### Effect of Intracellular Ions on Responses to ATP

**FIGURE 4.**
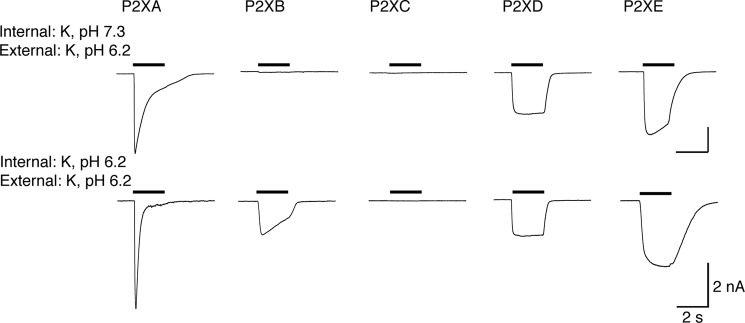
**ATP activates P2XB receptors when intracellular (≈cytoplasmic) solution is acidified.** Each *trace* shows membrane current during application of ATP (3 mm, 2 s: indicated by *bars*). Intracellular (≈cytoplasmic) cation was potassium. P2XA and P2XD receptors are unaffected by intracellular acidification. P2XB and P2XE currents are increased by acidification.

The intracellular or pipette solution in the present experiments corresponds to the cytoplasmic or intracellular solution in amoeba. The main cytoplasmic cation would be K^+^, and we therefore examined the actions of ATP with recording electrodes containing KCl. At pH 7.3, for all five P2X receptors, ATP-elicited currents were not significantly different whether the recording electrode contained Na^+^ or K^+^ (Figs. 2 and 4 and Table 1). However, when the recording electrodes contained K^+^ at pH 6.2, ATP (3 mm) evoked currents at P2XB receptors that were not observed under any other conditions. These currents declined to 0.55 ± 0.14 (*n* = 10) of their peak amplitude during a 2-s application of ATP.

**FIGURE 5.**
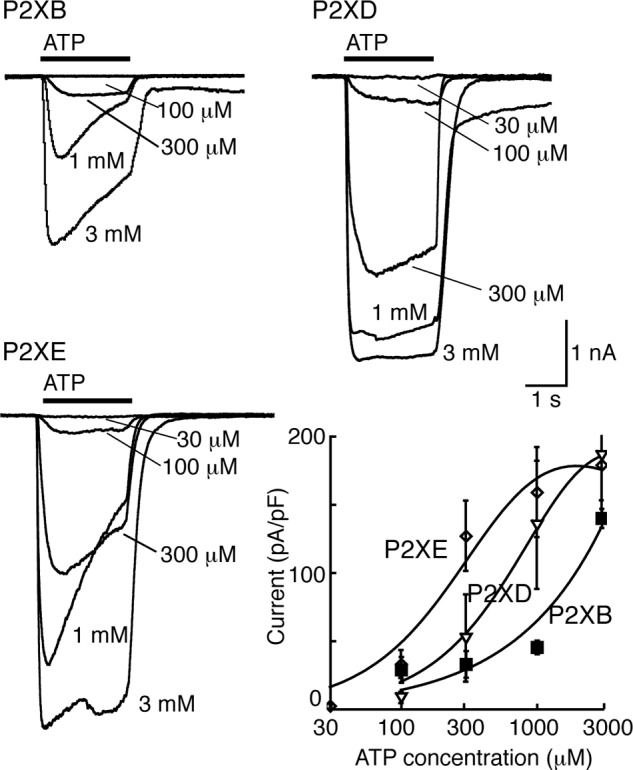
**Optimal conditions for ATP action at P2XB, P2XD, and P2XE receptors.** *Upper left*, currents evoked by ATP (2 s: indicated by *bar*) at P2XB receptors. External solution, potassium (pH 6.2); internal solution, potassium (pH 6.2). *Upper right*, currents evoked by ATP (2 s: indicated by *bar*) at P2XD receptors. External solution, potassium (pH 7.3); internal solution, potassium (pH 6.2). *Lower left*, currents evoked by ATP (2 s: indicated by *bar*) at P2XE receptors. External solution, potassium (pH 6.2); internal solution, potassium (pH 6.2). *Lower right*, concentration-response curves for the conditions indicated in the *other three panels*.

The results presented above, and summarized in Table 1, indicate the optimal ionic conditions for each P2X receptor when 3 mm ATP is used as the agonist. We therefore constructed corresponding concentration-response curves. Fig. 5 illustrates typical currents evoked by ATP at P2XB, P2XD, and P2XE receptors under these optimal conditions. Fig. 2 shows similar results for P2XA receptors. A comparison of these results indicates that P2XB, P2XD, and P2XE are all less sensitive to ATP than P2XA receptors.

##### Phenotypic Rescue of P2XA-null Dictyostelium by Other P2X Receptors

The preceding results indicate that all P2X receptors (except P2XC) are functional as ATP-activated ion channels, although they are optimally active under different conditions. However, because these assays were performed in a heterologous system we next sought evidence that they represent functional differences in living amoeba. For this, we tested whether differences in optimal ion channel activity compared with P2XA also reflected differences in the ability of each receptor to complement the function of P2XA. We have previously reported that P2XA-null mutant *Dictyostelium* cells show a prominent osmoregulatory defect in response to hypotonic shock (11). When wild type AX4 cells are transferred from a buffered salt solution (see “Experimental Procedures”) to water, they transiently swell over a 5–15-min period, but this is followed by a regulatory volume decrease (RVD). This process depends on fusion and discharge of contractile vacuoles, and by 30 min the cells have regained their original rather crenellated appearance. In contrast, P2XA-knock-out cells remain round or circular in appearance over 60 min, suggesting that they are less efficient at expelling water (11).

**FIGURE 6.**
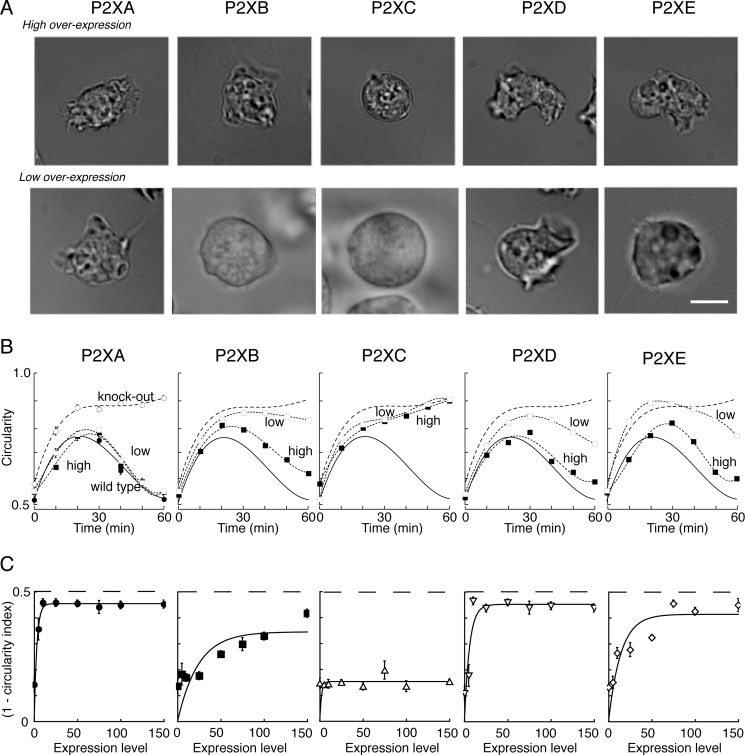
**Rescue of P2XA-null phenotype by other P2X receptors.** *A*, micrographs show *Dictyostelium* cells 40 min after changing the external solution to distilled water. *Left* to *right* shows P2XA-null cells that had been transfected with RFP-tagged P2X receptors, selected for a high (*top*) or low (*bottom*) level of RFP fluorescence. Wild type AX4 cells recover their normal crenelated shape at 40 min, and this was also observed with transfection of P2XA and P2XD receptors. *Scale bar* is 5 μm and applies to all *panels. B*, circularity increased as a function of time after placing cells in water. *Left panel* shows results for wild type (*filled circles*) and P2XA-null (*open circles*) cells. These lines are reproduced on the *other four panels*. All *panels* show the rescue (or not) of the wild type phenotype by transfection to low expression (*open squares*) or ∼5-fold higher expression (*filled squares*) levels, as judged by RFP fluorescence. Each point represents measurements from 30 to 55 cells. S.E. (*error bars*) is shown where it exceeds the size of the symbol. *C*, *ordinate* indicates the recovery of the wild type phenotype, expressed as (1 − circularity index) where circularity index is the ratio of mutually perpendicular cell diameters (shown is at 40 min). The value of (1 − circularity index) for AX4 cells was 0.35 ± 0.2 (*n* = 6) and for P2XA-null cells it was 0.16 ± 0.01 (*n* = 6) (*dashed line*). The *abscissa* shows the expression level of the transfected P2X receptor, measured as the intensity of RFP fluorescence (arbitrary units relative to cell area). Four to 19 cells were measured for each point.

Fig. 6 shows photomicrographs of typical cells taken 40 min after changing the extracellular solution to water: examples are shown for cells with low and high levels of expression. Overexpression of P2XA (tagged with RFP) restored the irregular, crumpled appearance of the *Dictyostelium* cells. The *lower left panel* of Fig. 6*C* illustrates that this occurs with very low levels of P2XA expression. Overexpression of P2XB, P2XD, and P2XE receptors also restored the capacity to recover from the hypotonic stimulus, with P2XD being most effective and able to restore RVD at low expression levels. In the case of P2XB the rescue was incomplete even at the highest expression levels, and P2XE also required greater expression levels compared with P2XA. P2XC overexpression failed to rescue the wild type phenotypic response to hypo-osmolarity (Fig. 6).

##### Is ATP the Most Effective P2X Receptor Ligand?

Using HEK cells expressing P2XA, and with Na^+^ as the predominant intracellular and extracellular cation, we tested several compounds for agonist activity. These were (all at 3 mm) ADP, AMP, cAMP, CTP, ITP, GTP, UTP, adenosine, cADPR, GMP, cGMP, UDP-glucose, NAD, reduced NAD (NADH), NADP, reduced NADP (NADPH), and NAADP. No compounds evoked significant currents. The only exception was ADP at P2XA receptors, which gave currents approximately 10% of the amplitude of those evoked by ATP. We also examined a mixture of nucleotides (CTP, GTP, UTP, ADP, AMP, and cAMP, each at 1 mm) on each of the five P2X receptors under all the recording conditions described above but in no case observed current greater than 50 picoamperes/picofarad. It is possible that these small currents result from the presence of ATP as an impurity in commercial preparations of other nucleotides.

The amino acid residues that make direct contacts with ATP in vertebrate P2X receptors are now known from extensive mutagenesis (20, 21) and from the crystal structure of an ATP-bound form of the zebrafish P2X4 receptor (15). Only a few of these residues are present at the corresponding positions of *Dictyostelium* P2X receptors, and this low level of conservation led us to wonder whether a ligand other than ATP might activate the receptors in the contractile vacuole of *Dictyostelium*. However, our search of 17 related compounds, many with known intracellular signaling roles, provided no evidence against the view that ATP is the preferred ligand at *Dictyostelium* receptors.

## DISCUSSION

Each of the five humanized *Dictyostelium* P2X receptors expressed well in HEK cells, as indicated by immunofluorescence at the plasma membrane (Fig. 1). Our findings from a systematic examination of their functional properties considerably extend the observations of Ludlow *et al.* (12), who used expression in *Xenopus* oocytes. This is because the HEK cell expression allows one to alter the ionic composition and pH of the intracellular (cytoplasmic in *Dictyostelium*) as well as extracellular (vacuolar in *Dictyostelium*) solutions. We found that P2XA, P2XB, P2XD, and P2XE exhibited robust currents in response to application of ATP in conditions where potassium was the predominant cation on both sides of the membrane and the pH on either side was 6.2 (Fig. 4, *lower trace*; Table 1, bottom line). P2XC receptors showed no response in this or in any other condition tested.

Using these “standard” conditions, we were able to assess which conditions increased or decreased the currents. For P2XA receptors, ATP-evoked currents were considerably larger than those observed for the other four receptors (Table 1), and changing the internal or external solution from K^+^ to Na^+^, or changing the internal or external pH from 6.2 to 7.3, had no significant effect on their peak amplitude. Although we did not observe any significant difference on peak currents, ATP was more effective at lower concentrations when extracellular K^+^ was replaced by Na^+^; this is consistent with the finding of Ludlow *et al.* (12) that P2XA currents were smaller when extracellular K^+^ was replaced by Na^+^. Likewise, changing pH did not alter peak currents when populations of cells were compared, but acidification of the internal or the external solution markedly increased desensitization (Figs. 2 and 4).

P2XB receptors showed significant ATP-evoked currents only under one condition tested. This was with K^+^ as the predominant cation on both sides of the membrane, and at acid pH (6.2) (Figs. 2 and 4 and Table 1). This result is broadly consistent with the report by Ludlow *et al.* (12), who found that currents at P2XB receptors were inhibited at pH 7.6 as well as by extracellular Na^+^. In the case of P2XD receptors, we observed currents only when the extracellular solution contained K^+^ rather than Na^+^, and these currents were inhibited by extracellular acidification (pH 6.2) (Table 1). Ludlow *et al.* (12) did not observe currents from P2XD receptors using oocytes, and this may be because they used an extracellular K^+^-based solution at pH 6.2. Our findings with P2XE receptors also correspond well with those of Ludlow *et al.* (12) in the sense that they can only be observed when the extracellular solution is K^+^ rather than Na^+^ (Table 1), but in addition we report that they are completely inhibited by raising the pH from 6.2 to 7.3.

From the perspective of the predominant cations surrounding the P2X receptors, these results can be summarized as follows. External Na^+^ (vacuolar in *Dictyostelium*) inhibits ATP-evoked currents somewhat at P2XA receptors, but profoundly at P2XB, P2XD, and P2XE receptors. This is consistent with observations in mammalian receptors, where external Na^+^ ions (compared with K^+^) have been found to inhibit currents at rat P2X2 receptors (22) and rat P2X7 receptors.^5^ Internal Na^+^ (cytoplasmic in *Dictyostelium*) inhibits ATP-evoked currents at P2XB receptors, but has little effect on ATP-evoked currents at P2XA, P2XD, and P2XE receptors.

From the perspective of protons, we find that ∼10-fold increase in external (vacuolar) H^+^ concentration has little effect on peak currents at P2XA receptors but accelerates their desensitization. It increases currents at P2XB and P2XE receptors, but inhibits currents at P2XD receptors. In comparison with mammalian P2X receptors, the variability is unsurprising given that currents at some P2X receptors are increased by lower extracellular pH whereas others are decreased (23, 24).

It would be desirable to interpret these results in the context of the actual ionic composition of the contractile vacuole, but here direct information is limited, and there is not much to add to Patterson's remark that “little is known of the nature of the fluid in contractile vacuoles” (25). The abundance of a proton pump, the V-type ATPase, is well established (4), particularly on the smaller tubules and swellings (spongiosome) of the contractile vacuole complex (26), and best estimates of vacuolar pH are in the region of 6.2 (3). It is also known that the contractile vacuole is relatively enriched in calcium (27), and Sivaramakrishnan and Fountain (13) have recently suggested that the vacuole functions as an intracellular calcium store. In the giant amoeba *Chaos carolinensis*, the vacuole is relatively enriched with sodium compared with the cytoplasm (for Na^+^, 20 mm in vacuole, 0.6 mm in cytoplasm; for K^+^, 5 mm in vacuole, 31 mm in cytoplasm) (28). These values serve to highlight the marked differences in intracellular osmolarity between these freshwater living amoeba and the mammalian cells used for expression in the present work and caution against any direct comparison of detailed properties.

When placed in ion-free water, *Dictyostelium* cells progressively swell over a period of several minutes and then undergo a regulatory volume decrease during which the contractile vacuole can be seen to reach the membrane and discharge its contents. In the *AX4* strain, this osmoregulation is markedly impaired in cells in which P2XA gene function has been disrupted (11). This defect can be corrected by overexpression of a P2XA-GFP construct in the P2XA-null cells. It has previously been shown that other *Dictyostelium* P2X receptors can also be found on the contractile vacuole when expressed from a constitutive promoter in vegetative growing cells (12). However, because we have discovered that although most can respond to ATP, each exhibits extremely different conditions favoring maximal responses, so we also asked whether such a phenotypic rescue could be effected by P2X receptors other than P2XA. The value for the circularity index (ratio of two perpendicular cell diameters) in wild type cells in normal KK2 solution was 0.65 ± 0.03 (*n* = 6), whereas cells became swollen and more rounded when placed in hypotonic solution, with the circularity index reaching 0.84 ± 0.04 (*n* = 6). Fig. 6*B* illustrates cells expressing even low levels of P2XA receptors, as judged by the independent measure of RFP fluorescence, showed a restoration of RVD: this essentially confirms our earlier finding (11). Overexpression of P2XD receptors also restored the osmoregulatory phenotype at relatively low levels of expression, whereas P2XB and P2XE receptors were less effective and required higher expression levels. Expression of P2XC receptors in the P2XA-null cells did not rescue the RVD, and the cells remained swollen at the 30-min observation period. The value of (1 − circularity index) depicted in Fig. 6*C* is not different from that observed in P2XA-null cells.

Together these findings support the idea that differences in optimal pH and ionic conditions for *Dictyostelium* P2X receptor function are likely to be functionally relevant and suggest that different receptors are unlikely to be functionally redundant. Furthermore, our findings provide a potential solution to how gating of intracellular P2X receptors can be achieved. Specifically, as ATP concentrations within cells are estimated to be in the region of several hundred micromolar it is more difficult to envisage how a localized source of ligand can be generated to allow temporal or spatial control of receptor activity. The finding that changes in pH or ionic condition can have such dramatic effects raises the possibility that changes in the intracellular conditions encountered by the receptor, rather than differences in ligand availability, could be an important regulator event. For example, P2XA and P2XD receptors may be poorly functional in the acidic environment of the vacuole: at pH 6.2 P2XA receptors showed prominent desensitization, and P2XD receptors did not respond to ATP. But as the bladder fills with water, the proton concentration would fall, and ATP would be better able to activate these two receptor types.

Finally, our data provide further support for the idea that activation of P2X receptors by ATP contributes to the normal response of this amoeba to hypotonic solution. The only one of the five receptors that failed to respond to ATP when expressed in HEK cells (P2XC) also failed to rescue the osmoregulatory phenotype in intact *Dictyostelium*. Our experiments have not addressed the mechanism by which P2X receptor activation contributes to contractile vacuole maturation, tethering, and water discharge. We note that Sivaramakrishnan and Fountain (13) have recently proposed that ATP transported into the vacuole acts on the P2X receptor, and this allows calcium to pass out into the cytosol and thereby initiate the docking process. The observation that P2X receptors with little or no calcium permeability (P2XB and P2XD) can also rescue the defect in our AX4 P2XA-null cells might suggest that functional channel(s) in *Dictyostelium* actually form as heterotrimers that incorporate two or three of the known subunits, or that other key interacting proteins (5, 6) which are missing from the heterologous expression system can critically alter the properties of the channel.

## Footnotes

4 K. Parkinson and C. R. L. Thompson, unpublished observations.

5 L.-H. Jiang and R. A. North, unpublished observations.

## Footnotes

3 The abbreviations used are:

RFP: red fluorescent protein
cADPR: cyclic adenosine diphosphate-ribose
HEK: human embryonic kidney
NAADP: nicotinic acid adenine dinucleotide phosphate
RVD: regulatory volume decrease.
